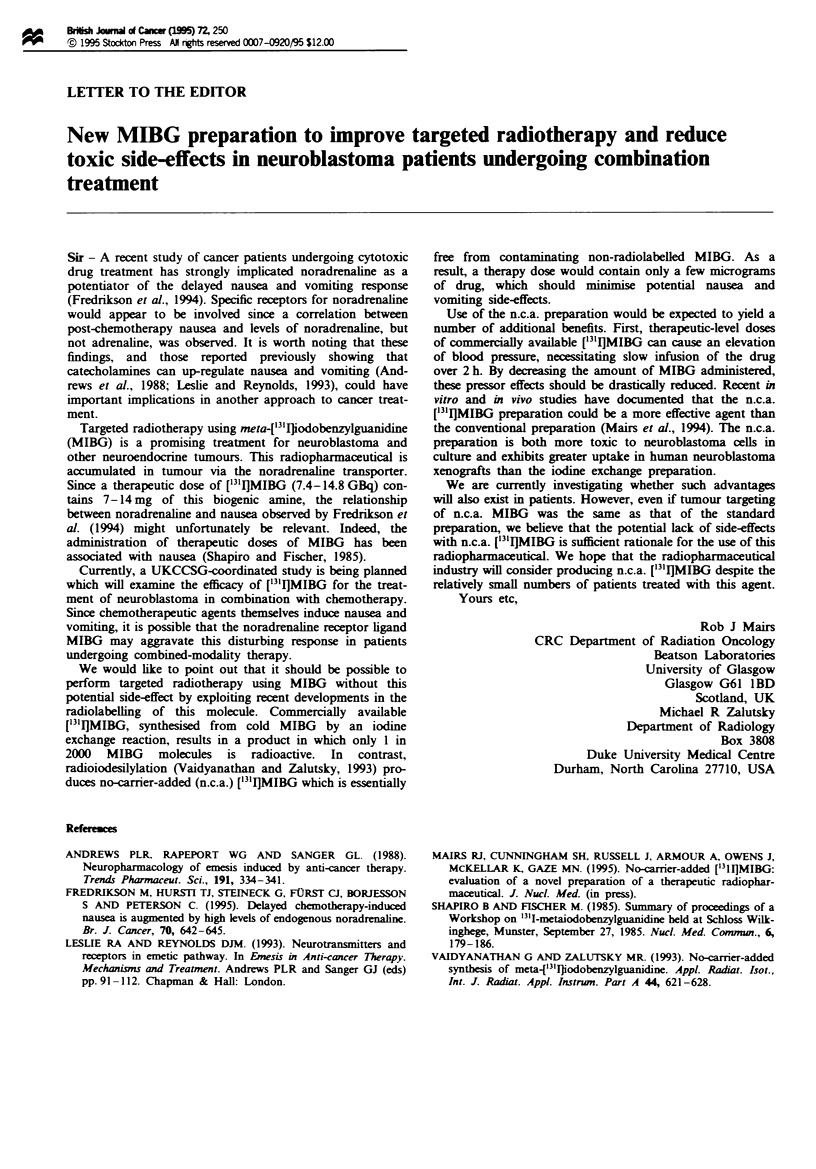# New MIBG preparation to improve targeted radiotherapy and reduce toxic side-effects in neuroblastoma patients undergoing combination treatment.

**DOI:** 10.1038/bjc.1995.312

**Published:** 1995-07

**Authors:** R. J. Mairs, M. R. Zalutsky


© 1995 Stockton Press All rights reserved 0007-0920/95 $12.00

LETTER TO THE EDITOR

### New MIBG preparation to improve targeted radiotherapy and reduce toxic side-effects in neuroblastoma patients undergoing combination treatment

Sir – A recent study of cancer patients undergoing cytotoxic drug treatment has strongly implicated noradrenaline as a potentiator of the delayed nausea and vomiting response (Fredrikson *et al.*, 1994). Specific receptors for noradrenaline would appear to be involved since a correlation between post-chemotherapy nausea and levels of noradrenaline, but not adrenaline, was observed. It is worth noting that these findings, and those reported previously showing that catecholamines can up-regulate nausea and vomiting (Andrews *et al.*, 1988; Leslie and Reynolds, 1993), could have important implications in another approach to cancer treatment.

Targeted radiotherapy using *meta*-[$^{131}$I]iodobenzylguanidine (MIBG) is a promising treatment for neuroblastoma and other neuroendocrine tumours. This radiopharmaceutical is accumulated in tumour via the noradrenaline transporter. Since a therapeutic dose of [$^{131}$I]MIBG (7.4–14.8 GBq) contains 7–14 mg of this biogenic amine, the relationship between noradrenaline and nausea observed by Fredrikson *et al.* (1994) might unfortunately be relevant. Indeed, the administration of therapeutic doses of MIBG has been associated with nausea (Shapiro and Fischer, 1985).

Currently, a UKCCSG-coordinated study is being planned which will examine the efficacy of [$^{131}$I]MIBG for the treatment of neuroblastoma in combination with chemotherapy. Since chemotherapeutic agents themselves induce nausea and vomiting, it is possible that the noradrenaline receptor ligand MIBG may aggravate this disturbing response in patients undergoing combined-modality therapy.

We would like to point out that it should be possible to perform targeted radiotherapy using MIBG without this potential side-effect by exploiting recent developments in the radiolabelling of this molecule. Commercially available [$^{131}$I]MIBG, synthesised from cold MIBG by an iodine exchange reaction, results in a product in which only 1 in 2000 MIBG molecules is radioactive. In contrast, radioiodesilylation (Vaidyanathan and Zalutsky, 1993) produces no-carrier-added (n.c.a.) [$^{131}$I]MIBG which is essentially free from contaminating non-radiolabelled MIBG. As a result, a therapy dose would contain only a few micrograms of drug, which should minimise potential nausea and vomiting side-effects.

Use of the n.c.a. preparation would be expected to yield a number of additional benefits. First, therapeutic-level doses of commercially available [$^{131}$I]MIBG can cause an elevation of blood pressure, necessitating slow infusion of the drug over 2 h. By decreasing the amount of MIBG administered, these pressor effects should be drastically reduced. Recent *in vitro* and *in vivo* studies have documented that the n.c.a. [$^{131}$I]MIBG preparation could be a more effective agent than the conventional preparation (Mairs *et al.*, 1994). The n.c.a. preparation is both more toxic to neuroblastoma cells in culture and exhibits greater uptake in human neuroblastoma xenografts than the iodine exchange preparation.

We are currently investigating whether such advantages will also exist in patients. However, even if tumour targeting of n.c.a. MIBG was the same as that of the standard preparation, we believe that the potential lack of side-effects with n.c.a. [$^{131}$I]MIBG is sufficient rationale for the use of this radiopharmaceutical. We hope that the radiopharmaceutical industry will consider producing n.c.a. [$^{131}$I]MIBG despite the relatively small numbers of patients treated with this agent.

Yours etc,

Rob J Mairs
CRC Department of Radiation Oncology
Beatson Laboratories
University of Glasgow
Glasgow G61 1BD
Scotland, UK
Michael R Zalutsky
Department of Radiology
Box 3808
Duke University Medical Centre
Durham, North Carolina 27710, USA

### References

ANDREWS PLR, RAPEPORT WG AND SANGER GL. (1988). Neuropharmacology of emesis induced by anti-cancer therapy. *Trends Pharmaceut. Sci.*, **191**, 334–341.

FREDRIKSON M, HURSTI TJ, STEINECK G, FÜRST CJ, BORJESSON S AND PETERSON C. (1995). Delayed chemotherapy-induced nausea is augmented by high levels of endogenous noradrenaline. *Br. J. Cancer*, **70**, 642–645.

LESLIE RA AND REYNOLDS DJM. (1993). Neurotransmitters and receptors in emetic pathway. In *Emesis in Anti-cancer Therapy. Mechanisms and Treatment*. Andrews PLR and Sanger GJ (eds) pp. 91–112. Chapman & Hall: London.

MAIRS RJ, CUNNINGHAM SH, RUSSELL J, ARMOUR A, OWENS J, MCKELLAR K, GAZE MN. (1995). No-carrier-added [$^{13}$1I]MIBG: evaluation of a novel preparation of a therapeutic radiopharmaceutical. *J. Nucl. Med.* (in press).

SHAPIRO B AND FISCHER M. (1985). Summary of proceedings of a Workshop on $^{131}$I-metaiodobenzylguanidine held at Schloss Wilkinghege, Munster, September 27, 1985. *Nucl. Med. Commun.*, **6**, 179–186.

VAIDYANATHAN G AND ZALUTSKY MR. (1993). No-carrier-added synthesis of meta-[$^{131}$I]iodobenzylguanidine. *Appl. Radiat. Isot., Int. J. Radiat. Appl. Instrum. Part A* **44**, 621–628.